# Cerebro-spinal fluid glucose and lactate concentrations changes in response to therapies in patIents with primary brain injury: the START-TRIP study

**DOI:** 10.1186/s13054-023-04409-6

**Published:** 2023-03-31

**Authors:** Elisa Gouvêa Bogossian, Chahnez Taleb, Raffaele Aspide, Rafael Badenes, Denise Battaglini, Federico Bilotta, Aaron Blandino Ortiz, Anselmo Caricato, Carlo Alberto Castioni, Giuseppe Citerio, Gioconda Ferraro, Costanza Martino, Isabella Melchionda, Federica Montanaro, Berta Monleon Lopez, Consolato Gianluca Nato, Michael Piagnerelli, Edoardo Picetti, Chiara Robba, Olivier Simonet, Aurelie Thooft, Fabio Silvio Taccone

**Affiliations:** 1 Department of Intensive Care, Erasme University Hospital, Université Libre de Bruxelles, Route de Lennik, 808, 1070 Brussels, Belgium; 2 Anesthesia and Neurointensive Care Unit, IRCCS Istituto Delle Scienze Neurologiche di Bologna, Via Altura, 3, Bologna, Italy; 3 Department of Anesthesiology and Surgical-Trauma Intensive Care, Hospital Clínic Universitari de Valencia, University of Valencia, Valencia, Spain; 4 Department of Surgical Science and Integrated Diagnostic, University of Genoa, Genoa, Italy; 5 IRRCS Policlinico San Martino, Genoa, Italy; 6 Department of Anaesthesiology, Critical Care and Pain Medicine, Umberto I Policlinico Di Roma, Rome, Italy; 7 Department of Intensive Care Unit, Ramón y Cajal University Hospital, Madrid, Spain; 8 Intensive Care Unit, Department of Anesthesiology and Intensive Care Medicine, Gemelli Hospital, Sacro Cuore Catholic University, Rome, Italy; 9 Scuola di Medicina e Chirurgia, Azienda Socio Sanitaria Territoriale Monza, Università Milano Bicocca, Monza, Italy; 10 Anesthesia and Intensive Care Unit, Azienda Romagna, M. Bufalini Hospital, Cesena, Italy; 11 Department of Intensive Care, CHU-Charleroi, Université Libre de Bruxelles, Charleroi, Belgium; 12 Experimental Medicine Laboratory, CHU-Charleroi, Montigny-Le-Tilleul, Belgium; 13 Department of Anesthesia and Intensive Care, Parma University Hospital, Parma, Italy; 14 Department of Anaesthesia and Intensive Care, Centre Hospitalier de Wallonie Picarde, Tournai, Belgium

**Keywords:** Acute brain injury, Subarachnoid hemorrhage, Traumatic brain injury, Intracerebral hemorrhage, Lactate, Glucose, Cerebrospinal fluid, Intracranial pressure

## Abstract

**Introduction:**

Altered levels of cerebrospinal fluid (CSF) glucose and lactate concentrations are associated with poor outcomes in acute brain injury patients. However, no data on changes in such metabolites consequently to therapeutic interventions are available. The aim of the study was to assess CSF glucose-to-lactate ratio (CGLR) changes related to therapies aimed at reducing intracranial pressure (ICP).

**Methods:**

A multicentric prospective cohort study was conducted in 12 intensive care units (ICUs) from September 2017 to March 2022. Adult (> 18 years) patients admitted after an acute brain injury were included if an external ventricular drain (EVD) for intracranial pressure (ICP) monitoring was inserted within 24 h of admission. During the first 48–72 h from admission, CGLR was measured before and 2 h after any intervention aiming to reduce ICP (“intervention”). Patients with normal ICP were also sampled at the same time points and served as the “control” group.

**Results:**

A total of 219 patients were included. In the intervention group (*n* = 115, 53%), ICP significantly decreased and CPP increased. After 2 h from the intervention, CGLR rose in both the intervention and control groups, although the magnitude was higher in the intervention than in the control group (20.2% vs 1.6%; *p* = 0.001). In a linear regression model adjusted for several confounders, therapies to manage ICP were independently associated with changes in CGLR. There was a weak inverse correlation between changes in ICP and CGRL in the intervention group.

**Conclusions:**

In this study, CGLR significantly changed over time, regardless of the study group. However, these effects were more significant in those patients receiving interventions to reduce ICP.

**Supplementary Information:**

The online version contains supplementary material available at 10.1186/s13054-023-04409-6.

## Introduction

Acute brain injury (ABI), such as traumatic brain injury (TBI), intracranial hemorrhage (ICH) and subarachnoid hemorrhage (SAH), is a significant cause of morbidity and mortality worldwide [1–3]. The complex pathophysiology responsible for secondary brain injury involves both systemic complications (i.e., hypoxemia, hypocapnia, fever, anemia, hyponatremia, hyperglycemia, etc.) [4–6], as well as cerebral complications, such as reduced cerebral perfusion pressure (CPP), cerebral edema, and blood–brain barrier dysfunction, tissue hypoxia, microvascular abnormalities, seizures and oxidative stress [7–9], all being associated with in an increased probability of poor prognosis.

In this setting, energetic metabolism is also often disturbed [10]. The injured brain may present with a reduced capacity to adequately utilize glucose as the primary source of fuel due to an impaired glucose transport to the brain tissue, which may have potential consequences on cellular viability and vulnerability to secondary insults [11]. Interestingly, studies using positron emission tomography (PET) scan and/or cerebral microdialysis (CMD) after ABI have also demonstrated that low extracellular glucose levels in the brain may result from an excessive glycolysis, in the absence of increased tissue perfusion and irrespective of systemic glucose concentrations [12–14].

In this setting, high cerebral extracellular lactate concentrations can be observed. Although initially considered a sign of anaerobic metabolism and tissue hypoxia, this phenomenon could also happen because of enhanced lactate uptake from the circulation or increased lactate production in the astrocytes [15–19], with a switch from glucose to lactate as the primary metabolic substrate for neuronal metabolism [20]. As such, low cerebral glucose and high cerebral lactate levels might indicate an energetic distress in ABI patients.

However, CMD and PET scans are not widely available and not feasible in many critically ill patients, while analyses of glucose and lactate concentrations in the cerebrospinal fluid (CSF) are more easily performed. A reduced cerebral spinal fluid glucose-to-lactate ratio (CGLR) has been associated with an increased risk of mortality and poor neurological recovery after ABI [21, 22]. However, it remains unclear whether CGLR is just a marker of severity or could be influenced by specific therapies.

As such, the objective of this study was to determine whether therapeutic interventions aiming to reduce intracranial pressure (ICP) would also result in CGLR changes in severe ABI patients.

## Methods

### Study population

This prospective multicentric observational study includes all patients admitted over 3 years following an aneurysmal SAH, TBI, ICH or other forms of ABI in 12 European Intensive Care Units (ICU). Patients were eligible for the study if they met the following criteria: (a) age > 18 years; (b) presence of an external ventricular drain (EVD) for intracranial pressure monitoring inserted within 24 h from admission. The local Ethics Committees approved this study in each participating center. According to local legislation, written informed consent for study participation was obtained from a patient family member or a legal representative. This study followed the recommendations of the Strengthening the reporting of observational studies in epidemiology (STROBE) guidelines [23].

### Patient management

Patients' management followed the current guidelines for the management of TBI [24], SAH [25] and ICH [26]. All patients also received “tier 0” therapy for intracranial hypertension [27]. The attending physician took the decision to initiate an intervention to reduce ICP, as well as the type of intervention, independently from the study protocol.

### Data collection

Patients’ demographics and pre-injury comorbid diseases were recorded. Clinical status on admission was evaluated using the Glasgow Coma Scale (GCS) [28]. The tomographic severity of the initial injury was assessed, according to the underlying disease, using the Marshall Score [29], the Fisher score [30] or the volume of ICH (> 30 ml). ICU mortality and the Glasgow Outcome Scale (GOS) [31] at 3 months were also reported, either collected from the medical charts or via the general practitioner. An unfavorable neurological outcome was defined as a GOS of < 4.

### Interventions

A CSF sample of 5 mL was collected from the proximal port of the EVD catheter using a sterile technique and analyzed within 60 min from the collection for biochemistry and cytology. CSF total counts of red and white blood cells, as well as protein concentrations, were obtained whenever possible before the start of any intervention; the first CSF lactate, CSF glucose, and CGLR assessment (i.e., with glucose and lactate both expressed in mmol/L) occurred within the first 72 h from ICU admission (i.e., “baseline”). In patients with ICP values requiring a specific therapy (“intervention” group), baseline measurement occurred just before the intervention and the second CSF sample was collected 2 h thereafter. In patients with relatively normal ICP values (“control” group), the baseline sample was also collected within 72 h from admission, according to the decision of local investigators, and the second sample 2 h thereafter, as in the intervention group. CSF glucose was measured using the hexokinase method; CSF lactate was measured using the enzymatic method in which L-lactate is oxidized to pyruvate and hydrogen peroxidase by lactate oxidase. However, each center had its own analyzer, reference values and internal validation procedures.

Blood gas analyses were performed at the same points, and arterial pH, PaCO_2_, PaO_2_, blood lactate and blood glucose levels were collected. Physiological variables, such as ICP, mean arterial pressure (MAP) and body temperature, were measured in real time and collected prospectively at the same time points. CPP was calculated as the difference between MAP and ICP. Vasopressors and insulin use, but not drug doses, were also recorded concomitantly with CSF samples.

Therapeutic interventions used to reduce ICP were then classified as “tier 1” (i.e., CSF drainage, increased sedation, and osmotic therapy with either hypertonic saline and/or mannitol) or “tier 2/3” (i.e., hyperventilation aiming at PaCO_2_ < 35 mmHg; barbiturate therapy, decompressive craniectomy, hypothermia or a combination of these strategies).

We also calculated the ΔICP, defined as the difference between ICP values at 2 h minus the value at baseline; similarly, the ΔCSF glucose, ΔCSF lactate and ΔCGLR were also calculated. The relative change in CGLR between the two different time points was also estimated for each patient as ([ΔCGLR/CGLR measured at baseline] *100). An “increase” in CGLR was defined as ΔCGLR > 0.

### Outcomes

The primary outcome of the study was the difference in ΔCGLR between the two groups. Secondary outcomes included: (a) the effects of the type of therapy (i.e., in particular CSF drainage vs others, according to the results of a pilot study—see below) on ΔCGLR; (b) the association of ICU mortality and unfavorable neurological outcome (UO) at 3 months on CGLR and ΔCGLR.

### Sample size

This was an exploratory study. An initial cohort of 21 patients was studied as a pilot phase to assess the feasibility of the two measurements, which showed that the CGLR was reduced by 15% among patients receiving an intervention, remaining almost unchanged in control patients. As such, to obtain a significant difference in ΔCGLR between the two groups, a total of 60 patients would be needed (power 90%, β-error 0.05). However, considering that interventions might provide different effects on ΔCGLR and to avoid bias in recruiting control patients, a cohort of at least 150 patients was considered adequate to evaluate the study hypothesis.

### Statistical analysis

Descriptive statistics were computed for all study variables. A Kolmogorov–Smirnov test was used, and histograms and normal-quartile plots were examined to verify the normality of the distribution of continuous variables. Data were presented as count (percentage), mean (± standard deviation) or median [25^th^–75^th^ percentiles], as appropriate. Differences between the two groups (intervention vs controls; survivors vs non-survivors and favorable vs unfavorable neurological outcome) were assessed using a chi-square or Fisher’s exact test for categorical variables and a t test (normally distributed variables) or a Mann–Whitney U test (independent nonparametric data) or Wilcoxon signed-rank test (nonparametric related data) for continuous variables. To account for repeated measures, ΔCGLR in the two groups were assessed using a mixed linear model, which considered the time (baseline vs. 2 h) and group (intervention vs. control) in the final analysis, both as categorical variables. A similar model was applied for other physiological and CSF variables. Univariable and multivariable linear models were constructed to assess the association of baseline variables and the percentage of ΔCGLR.

The discriminative ability of the CGLR at baseline to predict poor outcomes was evaluated using receiver operating characteristic (ROC) curves, with the corresponding area under the curve (AUROC). Youden’s index was computed to assess the optimal cutoff of the CGLR at baseline value for sensitivity and specificity to predict poor outcomes. Logistic regression analyses adjusted on the age, underlying pathology and GCS score and group were performed to assess whether CGLR at baseline was independently associated with mortality or unfavorable neurological outcome. In all multivariable models, collinearity between variables was excluded before modeling. A *p* < 0.05 will be considered statistically significant. Statistical analyses will be performed using IBM SPSS Statistics 28.0 for Macintosh.

## Results

### Study population

During the study period, 657 adult patients were admitted to the participating ICUs due to an ABI requiring EVD monitoring and were screened for inclusion; of those, 219 (33%) fulfilled the inclusion criteria and were analyzed (Additional file 1: Table S1). The most frequent etiology of brain injury was SAH (119/219, 54%), and the median GCS on admission was 8 (4–13) (Table 1). Of the 219 patients, 102 (47%) had CSF samples collected on day 1, 84 (38%) patients on day 2 and 33 (15%) patients on day 3. The overall mortality rate was 25% (55/219), and 51% of patients experienced unfavorable neurological outcomes at 3 months (111/219). The main physiological and CSF parameters of the study population are presented in Additional file 1: Table S2.

**Table 1:** Characteristics of the study population

	All patients (*N* = 219)	Controls (*N* = 104)	Intervention (*N* = 115)	*p* value
Age, years	57 (± 14)	59 (± 15)	55 (± 13)	0.07
Male gender, n (%)	112 (51)	49 (47)	63 (55)	0.28
GCS on admission	8 (4–13)	9 (5–13)	7 (4–12)	0.09
*Etiology, n (%)*				0.16
SAH	119 (54)	51 (49)	68 (59)	0.13
ICH	73 (33)	42 (40)	31 (27)	
TBI	25 (11)	10 (10)	15 (13)	
Others	2 (1)	1 (1)	1 (1)	
*Comorbidities, n (%)*				
Arterial Hypertension	116 (56)	56 (57)	60 (55)	0.78
Diabetes mellitus	33 (16)	14 (14)	19 (17)	0.57
Heart disease	33 (16)	12 (12)	21 (19)	0.19
COPD	17 (8)	8 (8)	9 (8)	0.99
Liver Cirrhosis	8 (4)	6 (6)	2 (2)	0.15
Chronic kidney disease	10 (5)	6 (6)	4 (4)	0.52
Previous neurological disease	21 (10)	9 (9)	12 (11)	0.82
Malignancies	16 (8)	10 (10)	6 (6)	0.30
Immunosuppression	7 (3)	4 (4)	3 (3)	0.71
EVD placement to sample collection, days	1 (1–2)	2 (1–2)	1 (1–2)	0.50
Admission to sample collection, days	2 (1–2)	2 (1–2)	1 (1–2)	0.50
*Outcomes*				
ICU length of stay, days	18 (12–25)	17 (11–25)	18 (13–27)	0.12
ICU mortality, n (%)	55 (25)	15 (14)	40 (35)	0.001
GOS at discharge	3 (2–4)	3 (3–4)	3 (1–3)	0.001
GOS at 3 months	3 (1–4)	4 (2–5)	3 (1–4)	0.002

Data are presented as mean (± SD), median (IQRs) and count (%), as appropriate

*GCS* Glasgow Coma Scale; *GOS* Glasgow Outcome Scale; *ICU* intensive care unit; *EVD* external ventricular drain; *COPD* chronic obstructive pulmonary disease

### Intervention vs. control groups

**Fig. 1:**
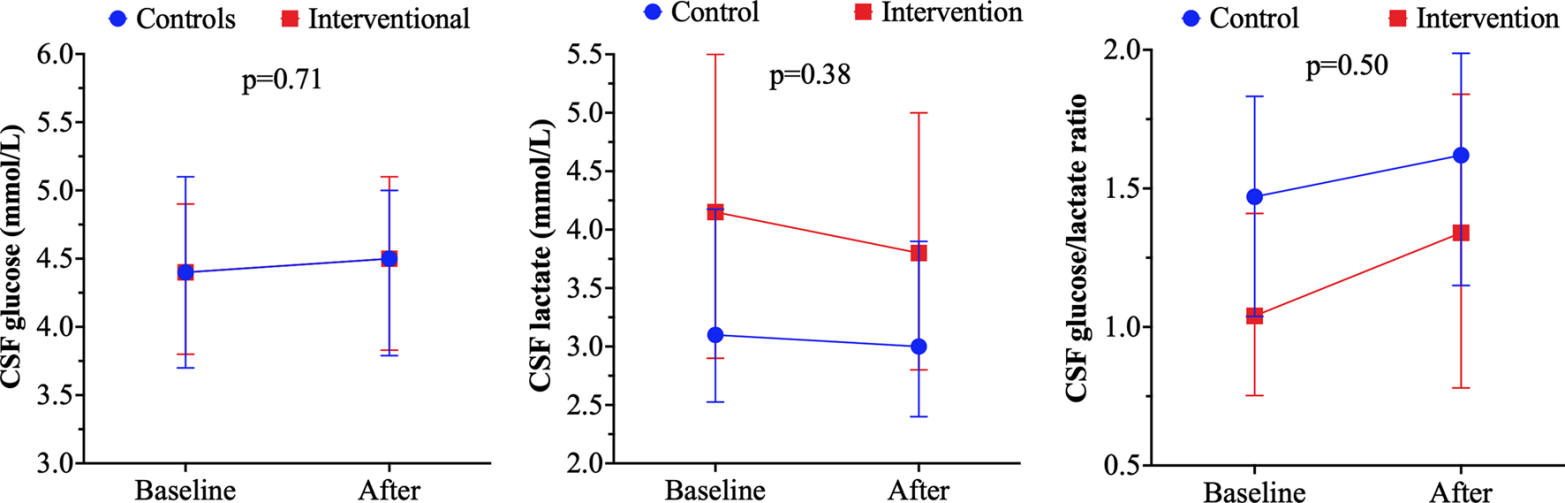
Changes in the cerebral spinal fluid glucose, lactate and glucose-to-lactate ratio (CGLR) over time (baseline and 2 h after) in the control and the interventional group. P values represent the comparison between the trend over time of the control and intervention groups (time-group interaction); *p* values were calculated using a mixed model

The characteristics of patients according to the study group are presented in Table 1. Patients in the intervention group (*n* = 115, 53%) were younger than controls; the ICU mortality rate was higher in the intervention group when compared to controls, as well as the rate of patients with the unfavorable neurological outcome at 3 months. Patients in the intervention group had higher ICP values and lower CGLR values at baseline and at 2 h, when compared with the control group (Additional file 1: Table S3). An increase in CGLR after 2 h was observed in 80 (70%) patients in the intervention group and 59 (57%) in the control group (*p* = 0.05). In the intervention group, CGLR was increased by 20.2% (95% CI from −20.2 to 54.9%), when compared to an increase of 1.6% (95% CI from −6.7 to 15.9%; *p* = 0.001) in the control group. Table 2 and Fig. 1 show the comparison between groups of the trend of CSF and physiological variables over time (i.e., time-group interaction). In the linear regression analysis (Additional file 1: Table S4) adjusted for GCS, ABI etiology, baseline ICP and baseline CGLR, the intervention compared to the control group was independently associated with a higher percentage of increase in $\Delta {\text{CGLR}}$ at 2 h (beta coefficient 27.47 [95% CI 11.71–43.23]; *p* = 0.001).

**Table 2:** Comparison of changes in physiological and cerebral spinal fluid (CSF) variables according to study group

	Control group		Intervention group		*p* Value (time*group)
	Baseline	2 h	Baseline	2 h	
Temperature, °C	36.8 (36.2–37.4)	36.8 (36.3–37.2)	36.8 (36.3–37.4)	37.0 (36.5–37.5)	0.79
MAP, mmHg	92 (85–104)	91 (86–104)	96 (89–104)	97 (88–103)	0.86
ICP, mmHg	9 (6–12)	8 (6–11)	21 (15–25)	14 (9–18)	0.001
CPP, mmHg	81 (76–93)	83 (75–95)	77 (67–86)	83 (72–90)	0.07
PaCO2, mmHg	38 (36–42)	38 (35–40)	37 (35–40)	37 (35–40)	0.29
Hb, g/dL	11.9 (10.8–12.9)	11.8 (10.5–12.6)	11.3 (10.4–12.7)	11.3 (10.2–12.4)	0.97
CSF RBC, 10^3^/mm^3^	11.2 (2.0–50.6)	12.1 (1.8–68.3)	30.5 (3.1–88.4)	22.5 (3.6–93.9)	0.74
CSF WBC, /mm^3^	31 (10–140)	35 (12–158)	101 (24–342)	105 (24–380)	0.73
CSF Proteins, mg/dL	72 (50–160)	76 (46–153)	98 (63–180)	85 (57–167)	0.85
CSF glucose, mg/dL	79 (66–92)	81 (69–92)	79 (68–89)	80 (68–90)	0.71
Blood glucose, mg/dL	142 (119–154)	138 (118–152)	143 (129–157)	139 (126–164)	0.65
Glucose CSF/blood	0.59 (0.51–0.67)	0.61 (0.48–0.65)	0.55 (0.46–0.64)	0.57 (0.49–0.66)	0.31
CSF lactate, mEq/L	3.1 (2.6–4.2)	3.0 (2.4–3.9)	4.2 (2.9–5.5)	3.8 (2.8–5.0)	0.38
Blood lactate, mEq/L	1.0 (0.8–1.3)	1.0 (0.8–1.2)	1.2 (0.9–1.8)	1.0 (0.8–1.3)	0.84
Lactate CSF/blood	3.0 (2.0–4.1)	3.0 (2.1–4.1)	3.2 (2.3–4.6)	3.6 (2.6–5.0)	0.27
CGLR	1.47 (1.04–1.83)	1.62 (1.15–1.98)	1.04 (0.76–1.41)	1.34 (0.80–1.83)	0.50

Data are presented as median (IQR), unless otherwise specified

*CGLR* cerebral spinal fluid glucose-to-lactate ratio; *CSF* cerebral spinal fluid; *RBC* red blood cells; *WBC* white blood cells; *MAP* mean arterial pressure; *ICP* intracranial pressure; *CPP* cerebral perfusion pressure; *PaCO*_*2*_ arterial partial pressure of carbon dioxide; *Hb* hemoglobin

P values represent the comparison of changes over time of the two groups (time-group interaction) and were calculated using a mixed model

**Fig. 2:**
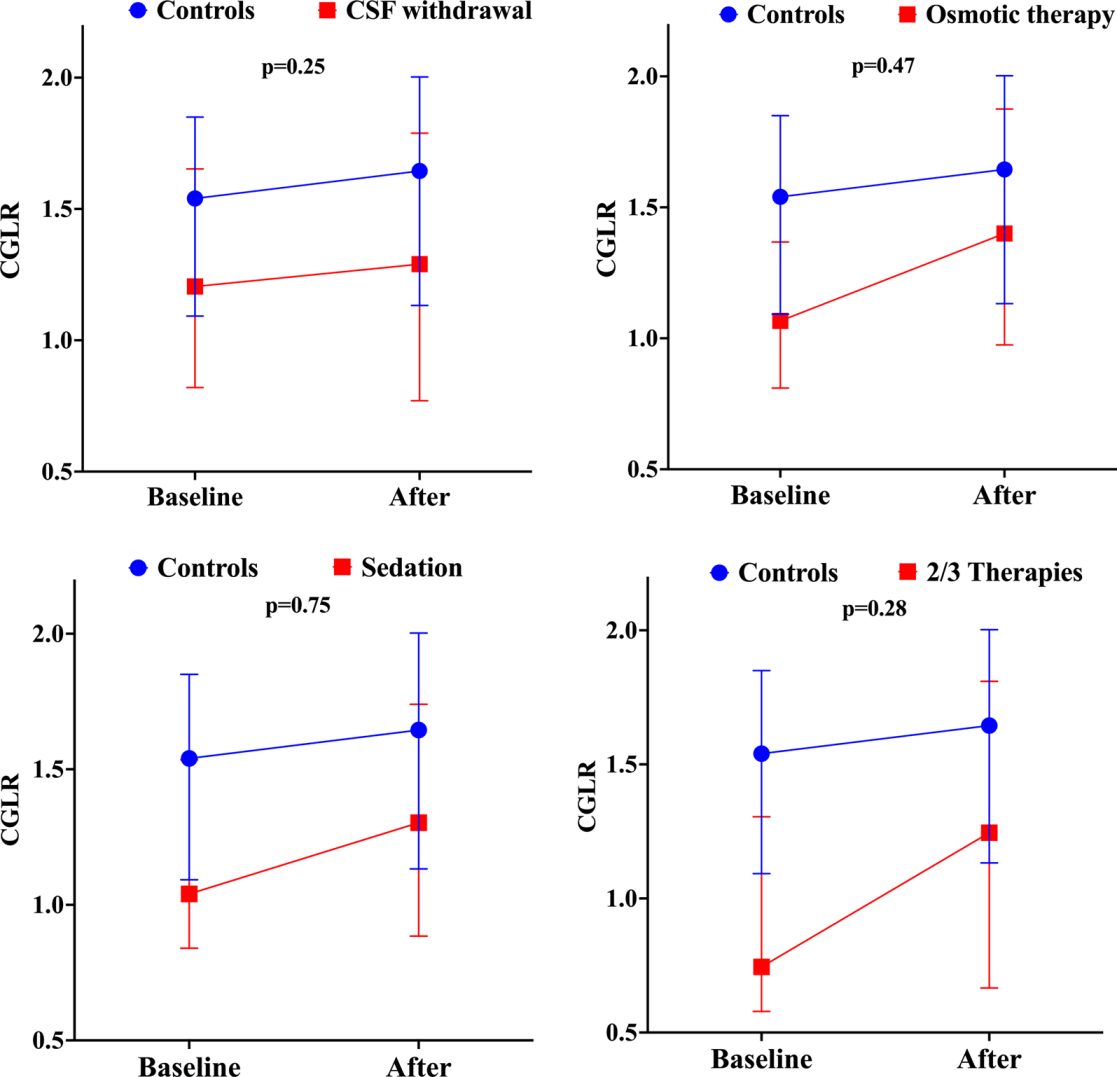
Comparison of the changes in the cerebral spinal fluid glucose-to-lactate ratio (CGLR) over time in the control and different intervention subgroups. *p* values represent the comparison between trend over time of the two groups (time-group interaction) and were calculated using a mixed model

In the intervention group, ICP significantly decreased after treatment, while CPP increased when compared to baseline (Table 3); CGLR increased after the intervention, while CSF lactate levels decreased (Table 3). The most common strategy used to reduce ICP was osmotic therapy with either hypertonic saline or mannitol (53/115, 46%), followed by CSF drainage (45/115, 39%); tier 2/3 therapies were used in 18/115 patients (16%). ICP significantly decreased and CPP increased in all intervention subgroups (Table 3). In the osmotic therapy subgroup, CSF lactate and CSF glucose decreased after 2 h, while CGLR significantly increased (Fig. 2); CGLR was increased by 6.1% (95% CI from −5.4 to 32.2%). In the CSF drainage and the sedation subgroup, CGLR also increased after 2 h, although this was not statistically significant when compared to baseline values; CGLR was increased by 37.5% (95% CI from −2.6% to 85.1%) and 19.4 (95% CI from −11.1 to 41.9), respectively (Fig. 2). In the tier 2/3 subgroup, CSF lactate significantly decreased and CGLR significantly increased; CGLR was increased by 21.7% (95% CI from 6.9 to 43.7%—Fig. 2).

**Table 3:** Physiological and cerebral spinal fluid (CSF) data of the interventional and control groups at two time points (baseline and at 2 h)

	All interventions (*N* = 115)			Osmotic therapy (*N* = 53)			CSF withdrawal (*N* = 45)			Sedation (*N* = 13)			Tier 2/3 (*N* = 18)			Controls (*N* = 104)		
	T0	T2	*p* Value	T0	T2	*p* Value	T0	T2	*p* Value	T0	T2	*p* Value	T0	T2	*p* Value	T0	T2	*p* Value
ICP, mmHg	21 (15–25)	14 (9–18)	0.001	23 (20–26)	15 (9–18)	0.001	15 (12–19)	13 (8–14)	0.001	24 (21–25)	13 (11–18)	0.001	27 (22–36)	18 (12–22)	0.001	9 (6–12)	8 (6–11)	0.66
CPP, mmHg	77 (67–86)	83 (72–90)	0.001	73 (62–80)	79 (70–90)	0.01	84 (74–88)	85 (76–91)	0.04	73 (65–84)	79 (67–87)	0.05	69 (55–83)	79 (67–87)	0.02	81 (76–93)	83 (75–95)	0.78
CSF Glucose, mg/dL	79 (68–89)	80 (68–90)	0.25	81 (70–94)	80 (68–90)	0.02	77 (67–85)	80 (68–90)	0.53	86 (78–86)	83 (65–88)	0.62	83 (71–90)	81 (69–92)	0.87	79 (66–92)	81 (69–92)	0.20
CSF lactate, mEq/L	4.2 (2.9–5.5)	3.8 (2.8–5.0)	0.001	4.1 (2.9–5.2)	3.6 (2.7–4.6)	0.02	3.9 (2.8–5.0)	3.7 (2.8–4.8)	0.89	4.7 (3.2–5.5)	3.6 (2.9–5.1)	0.04	5.5 (4.1–7.2)	4.9 (3.0–6.6)	0.05	3.1 (2.6–4.2)	3.0 (2.4–3.9)	0.05
CGLR	1.04 (0.76–1.41)	1.34 (0.80–1.83)	0.001	1.01 (0.79–1.28)	1.40 (0.97–1.87)	0.001	1.20 (0.83–1.63)	1.29 (0.78–1.77)	0.49	0.98 (0.81–1.49)	1.03 (1.02–1.58)	0.12	0.74 (0.57–1.27)	1.03 (0.66–1.78)	0.002	1.47 (1.04–1.83)	1.62 (1.15–1.98)	0.02

Data are presented as median and interquartile range (IQR). T0 represents baseline and T2 represents 2 h after

*CGLR* cerebral spinal fluid glucose-to-lactate ratio; *CSF* cerebral spinal fluid; *ICP* intracranial pressure; *CPP* cerebral perfusion pressure

*P* value was calculated using the Wilcoxon signed-rank test for related samples

In the control group, there was no significant difference in ICP, CSF glucose levels between baseline and after 2 h. CSF lactate levels significantly decreased, and CGLR increased after 2 h when compared to baseline (Table 3).

### CGLR, mortality and neurological outcome

The characteristics of the patients according to mortality and neurological outcome are shown in Additional file 1: Tables S5 and S6; CGLR at baseline were significantly lower in non-survivors compared to survivors. In a multivariable model (Additional file 1: Table S7) adjusted for age, ABI etiology and GCS on admission, baseline CGLR was independently associated with ICU mortality (OR 0.34 [95% CI 0.18–0.65]). The lower the CGLR level at baseline, the higher the probability of ICU mortality; the AUROC for the ability of CGLR at baseline to predict ICU mortality was 0.73 (95% CI 0.62–0.78: Additional file 1: Fig. S1), with an optimal cutoff of 1.21 (Sensibility = 74% and Specificity = 62%).

Patients with unfavorable neurological outcomes had lower GCS on admission, higher CSF lactate and lower CGLR at baseline, when compared to others. In a multivariable model (Additional file 1: Table S8) adjusted for age, ABI etiology and GCS on admission, baseline CGLR was independently associated with neurological outcome (OR 0.47 95% CI 0.29–0.75). The lower the CGLR, the higher the probability of unfavorable neurological outcome; the AUROC for the ability of baseline CGLR to predict adverse outcomes at 3 months was 0.66 (95% CI 0.59–0.74, Additional file 1: Fig. S2), with an optimal cutoff of 1.39 (Sensibility = 72% and Specificity = 58%).

### CGLR according to different etiologies

In TBI patients (*n* = 25), the changes in CGLR over time were not statistically significant in the control (baseline: 1.96 [1.28–2.42] vs. 2 h: 1.94 [1.48–2.35], *p* = 0.51) and the intervention (baseline: 1.04 [0.87–1.52] vs. 2 h: 1.35[1.00–1.77], *p* = 0.06] group. In ICH patients (*n* = 73), while in the control group CGLR did not significantly change over time (baseline: 1.56 [1.17–1.88] vs. 2 h: 1.60 [1.15–1.87], *p* = 0.59), a significant increase in CGLR (baseline: 1.0 [0.72–1.31] vs. 2 h: 1.28 [0.74–1.48], *p* = 0.02) was observed in the intervention group. In SAH patients (*n* = 119), CGLR significantly increased over time both in the control (baseline: 1.40 [0.98–1.73] vs. 2 h: 1.55 (1.11–2.02), *p* = 0.005] and the intervention (baseline: 1.07 [0.77–1.47] vs. 2 h: 1.43 [0.83–1.90], *p* = 0.001) groups. The trend of CGLR over time was similar between groups in TBI, ICH and SAH patients, as shown in Additional file 1^1^: Figs. S3–S5.

## Discussion

In the present study, we observed that the CSF glucose-to-lactate ratio increased in patients who received treatments to manage ICP as in those with stable ICP. However, a larger increase in CGLR was observed in the intervention group, when compared to controls, probably as an effect of ICP therapy (i.e., lower CGLR at baseline because of higher ICP and larger metabolic improvement when treatment was given). The administration of osmotic agents and tier 2/3 therapies significantly increased CGLR, while CSF drainage and sedation resulted in marginal CGLR changes. Finally, a lower CGLR at baseline was independently associated with ICU mortality and unfavorable neurological outcome at 3 months.

CSF analysis has an important role in the management of several infectious and non-infectious neurological conditions, as it provides information on the presence of blood, inflammation, infection as well as degenerative diseases [25, 32–35]. In acute brain injury patients, in whom an EVD has been inserted [36], CSF analysis is a readily available, easy-to-perform procedure to have important information on infectious complications [37], but also, as suggested by these findings, some insights on brain metabolism, with some prognostic value. [38] As a potential surrogate of anaerobic metabolism [21, 22], low CGLR should be further studied in these patients to better understand its feasibility (i.e., how many measurements per day and over the ICU stay), its clinical use (i.e., guide therapies or better stratify ICP severity) and potential limitations (i.e., correlated with microdialysis findings, false positive, cutoff to predict the need for interventions) in clinical practice.

To the best of our knowledge, this is the first study to address the impact of ICP-directed therapies on CGLR, although different studies have shown a decrease in the lactate-to-pyruvate ratio (LPR) measured by CMD when specific therapies to reduce ICP were given [13, 39]. We also observed that CGLR increased regardless of the administration of some therapeutic interventions over time. However, the increase in CGLR was significantly higher in the intervention group. These findings can have different explanations. First, CSF glucose and lactate require more time to respond to specific interventions when compared to CMD and the 2-h observation period was probably too short. However, this interval was selected to exclude additional interventions or events (i.e. shivering, transport, fluid administration, etc.) that might have influenced CGLR and were set according to each center’s clinical practice. Second, CSF glucose and lactate levels are also affected by plasma levels of glucose and lactate and are less reliable in assessing the metabolic status of brain parenchyma. In a previous study, CSF and blood levels of these two molecules showed only a modest correlation, while no studies have compared CSF and CMD levels of such molecules. Third, half of the patients in the intervention group did not present significant intracranial hypertension (i.e., ICP > 20–22 mmHg) at baseline, resulting in a less significant effect on brain metabolism of these therapeutic interventions; this may happen because of CSF was continuously drained to prevent ICP surge or because other triggers (i.e., low brain oxygenation values) could have been used to improve cerebral hemodynamics. Fourth, the effects on CGLR are largely dependent on the type of intervention. Indeed, CSF drainage only minimally impacts cerebral perfusion in the absence of intracranial hypertension or overt hydrocephalus. Conversely, osmotic therapy, sedatives and more aggressive interventions significantly influence brain hemodynamics and metabolism and were associated with a more considerable increase in CGLR. However, the limited number of patients receiving sedatives prevented more robust statistical analyses on this topic. As such, the study might have been underpowered to detect significant CGLR changes in therapeutical subgroups.

We believe that our findings have important clinical relevance. First CGLR assessment can identify patients with a more relevant brain injury, as suggested by the prognostic value of CGLR [17, 39]. This is in line with previous studies conducted with CMD, which have shown that elevated levels of cerebral lactate can be used to identify ischemia and anaerobic metabolism [40]. However, lactate levels can also increase despite adequate perfusion due to hyperglycolysis, neuro-inflammation and adrenergic stimulation [12, 41]. In this setting, the lactate-to-pyruvate ratio (LPR) better reflects the cellular redox state [42] and is a good marker of metabolic distress with or without concomitant ischemia [43, 44]. In the absence of pyruvate measurement in the CSF, glucose could be considered together with lactate levels; low CMD glucose levels can reflect energetic dysfunction and/or an hypoxic injury [45].

Regarding CSF analysis, Fujishima et al. demonstrated an increase in CSF lactate and LPR immediately after the injury, followed by a gradual reduction in the following weeks, especially in patients with unfavorable outcomes [46]; CSF lactate levels were higher in patients with unfavorable neurological outcomes than the others during the first days after the injury. Previous studies have shown that reductions in CGLR are independently associated with adverse outcomes in TBI and SAH patients [21, 22]. As such, increasing ICP values with concomitant high CGLR might be a clinically available trigger to administer ICP-directed therapies, individualizing therapeutic decisions rather than using a fixed ICP cutoff.

The present study has several limitations. First, we did not concomitantly collect data from CMD catheters and, therefore, could not compare the predictive value of glucose and lactate sampled using the two different techniques. Moreover, we did not measure pyruvate in the CSF, which can also be an interesting marker to be assessed. Secondly, we collected paired CSF glucose and lactate only once per patient; although the study did not focus on this issue, repeated CSF measurements could increase the risk of infections and it would be difficult to propose such daily strategy in ABI patients. Third, we did not evaluate the potential causes of low CGLR (i.e., high ICP, low CPP, cerebral vasospasm, seizures, ventriculitis, etc.) in our study cohort. Fourth, the delay between admission and CGLR assessment was not the same for all patients, which could have also impacted our results. Fifth, we included different acute brain injury etiologies with different pathophysiology which can have influenced our results. Sixth, we did not account for differences in the intensity of treatment throughout the ICU stay and specially in the first 72 h. Finally, CSF glucose and lactate measurements were not performed using the same analyzers; although this might potentially influence the absolute values, CGLR (as a ratio) and relative changes over time (as glucose and lactate would be measured on the same device) should be unaffected.

## Conclusions

In this study, CGLR increased over time in the two groups. These effects were more significant in those patients receiving ICP-directed therapies, in particular osmotics or tier 2/3 therapies. These findings also confirmed that low CGLR measured in the first 72 h after ABI was a marker of poor prognosis.

## Supplementary Information

**Additional file 1**. Supplemental electronic material of STAR-TRIP study.

## Data Availability

All data generated or analyzed during this study are included in this published article and its supplementary information files.
