# Supplementary material for: Cerebro-spinal fluid glucose and lactate concentrations changes in response to therapies in patIents with primary brain injury: the START-TRIP study
Source: Crit Care. 2023 Mar 31;27:130. doi: 10.1186/s13054-023-04409-6 (PMC10067218; doi:10.1186/s13054-023-04409-6)

**Supplemental Table S1:** Distribution of screened (i.e. adult patients with an acute brain injury requiring external ventricular device placement) and recruited patients, according to participating centers.

|  | **Screened** | **Excluded** | **Reasons for exclusion** | **Included**  **N=219** | **Intervention**  **N=115** | **Control**  **N=104** |
| --- | --- | --- | --- | --- | --- | --- |
| **Brussels** | 133 | 18 | EVD not draining: 8  Missed: 3  EVD implanted after 24 hours from admission: 7 | 115 | 61 | 54 |
| **Cesena** | 12 | 8 | EVD not draining: 2 patients  EVD positioned due to chronic hydrocephalus: 4  Missed: 2 | 3 | 1 | 2 |
| **Tournai** | 14 | 10 | EVD not draining: 1  Absent CSF samples:8  Bacterial Meningitis: 1 | 4 | 2 | 2 |
| **Genova** | 55 | 31 | No Informed Consent: 31 | 12 | 12 | 0 |
| **Monza** | 36 | 35 | Missed: 35 | 1 | 1 | 0 |
| **Bologna** | 32 | 13 | Brain Tumor: 5  No Informed Consent: 5  EVD implanted after 24 hours from admission: 3 | 19 | 2 | 17 |
| **Roma I** | 60 | 50 | Missed:5  No Informed Consent: 45 | 10 | 5 | 5 |
| **Roma II** | 183 | 176 | Early removal of EVD: 62  Missed: 43  No Informed Consent: 71 | 7 | 4 | 3 |
| **Madrid** | 36 | 13 | Imminent Death: 13 | 23 | 13 | 10 |
| **Ferrara** | 35 | 25 | Chronic Hydrocephalus: 10  Missed: 5  EVD not draining: 5  Absent CSF samples: 5 | 10 | 4 | 6 |
| **Charleroi** | 23 | 14 | No Informed Consent: 3  EVD installed after 72 hours from admission: 5  Absent CSF samples: 3  Imminent Death: 2  COVID-19: 1 | 9 | 6 | 3 |
| **Valencia** | 38 | 32 | Less than 18 years of age: 1  EVD inserted after 24 hours frm admission: 31 | 6 | 4 | 2 |

**Supplemental Table S2**: Main physiological and cerebrospinal fluid data of the study population at the two time-points. Data are presented as median and interquartile range (IQR) or count (%).

|  | **Baseline** | **2 hours** | **p-value** |
| --- | --- | --- | --- |
| Temperature, °C | 36.8 (36.3-37.4) | 36.9 (36.4-37.3) | 0.43 |
| MAP, mmHg | 94 (86-104) | 93 (86-103) | 0.65 |
| ICP, mmHg | 14 (8-22) | 11 (7-15) | 0.001 |
| CPP, mmHg | 79 (70-89) | 83 (74-92) | 0.001 |
| PaCO_2_, mmHg | 37 (35-41) | 37 (35-41) | 0.50 |
| Hb, g/dL | 11.6 (10.6-12.9) | 11.4 (10.4-12.6) | 0.001 |
| CSF RBC, 10^3^/mm^3^ | 15.2 (2.3-79.2) | 12.0 (2.0-54.0) | 0.03 |
| CSF WBC, /mm^3^ | 68 (13-220) | 73 (18-250) | 0.88 |
| CSF Proteins, mg/dL | 94 (55-170) | 80 (51-164) | 0.02 |
| CSF Glucose, mg/dL | 79 (67-89) | 80 (69-91) | 0.99 |
| Blood glucose, mg/dL | 142 (126-156) | 138 (122-155) | 0.55 |
| Glucose CSF/blood | 0.57 (0.48-0.66) | 0.59 (0.49-0.66) | 0.16 |
| CSF lactate, mEq/L | 3.5 (2.7-4.9) | 3.3 (2.6-4.5) | 0.14 |
| Blood lactate, mEq/L | 1.1 (0.8-1.5) | 1.0 (0.8-1.2) | 0.001 |
| Lactate CSF/blood | 3.1 (2.1-4.4) | 3.3 (2.4-4.5) | 0.07 |
| CGLR | 1.27 (0.85-1.68) | 1.45 (0.97-1.89) | 0.001 |
| Insulin, n (%) | 66 (30) | 67 (30) | 0.99 |
| Noradrenaline, n (%) | 117 (54) | 116 (53) | 0.99 |

CGLR: cerebral spinal fluid glucose to lactate ratio; CSF: cerebral spinal fluid; RBC: red blood cells; WBC: white blood cells; MAP: mean arterial pressure; ICP: intracranial pressure; CPP: cerebral perfusion pressure; PaCO_2_: arterial partial pressure of carbon dioxide; Hb: hemoglobin.

**Supplemental table S3:** Main physiological and cerebrospinal fluid data of the control and intervention groups at the two time-points. Data are presented as median and interquartile range (IQR) or count (%).

|  | **Control group**  **N= 104** | **Intervention group**  **N=115** | **p- value** |
| --- | --- | --- | --- |
| *Baseline* | | | |
| Temperature, °C | 36.8 (36.2-37.4) | 36.8 (36.3-37.4) | 0.43 |
| MAP, mmHg | 92 (85-104) | 96 (89-104) | 0.100 |
| ICP, mmHg | 9 (6-12) | 21 (15-25) | 0.001 |
| CPP, mmHg | 81 (76-93) | 77 (67-86) | 0.001 |
| PaCO_2_, mmHg | 38 (36-42) | 37 (35-40) | 0.12 |
| Hb, g/dL | 11.9 (10.8-12.9) | 11.3 (10.4-12.7) | 0.16 |
| CSF RBC, 10^3^/mm^3^ | 11.2 (2.0-50.6) | 30.5 (3.1-88.4) | 0.03 |
| CSF WBC, /mm^3^ | 31 (10-140) | 101 (24-342) | 0.002 |
| CSF Proteins, mg/dL | 72 (50-160) | 98 (63-180) | 0.09 |
| CSF Glucose, mg/dL | 79 (66-92) | 79 (68-89) | 0.56 |
| Blood glucose, mg/dL | 142 (119-154) | 143 (129-157) | 0.08 |
| Glucose CSF/blood | 0.59 (0.51-0.67) | 0.55 (0.46-0.64) | 0.03 |
| CSF lactate, mEq/L | 3.1 (2.6-4.2) | 4.2 (2.9-5.5) | 0.001 |
| Blood lactate, mEq/L | 1.0 (0.8-1.3) | 1.2 (0.9-1.8) | 0.01 |
| Lactate CSF/blood | 3.0 (2.0-4.1) | 3.2 (2.3-4.6) | 0.09 |
| CGLR | 1.47 (1.04-1.83) | 1.04 (0.76-1.41) | 0.001 |
| Insulin, n (%) | 32 (31) | 34 (30) | 0.88 |
| Noradrenaline, n (%) | 41 (39) | 70 (67) | 0.001 |
| *2 hours* | | | |
| Temperature, °C | 36.8 (36.3-37.2) | 37.0 (36.5-37.5) | 0.18 |
| MAP, mmHg | 91 (86-104) | 97 (88-103) | 0.12 |
| ICP, mmHg | 8 (6-11) | 14 (9-18) | 0.001 |
| CPP, mmHg | 83 (75-95) | 83 (72-90) | 0.18 |
| PaCO2, mmHg | 38 (35-40) | 37 (35-40) | 0.33 |
| Hb, g/dL | 11.8 (10.5-12.6) | 11.3 (10.2-12.4) | 0.17 |
| CSF RBC, 10^3^/mm^3^ | 12.1 (1.8-68.3) | 22.5 (3.6-93.9) | 0.07 |
| CSF WBC, /mm^3^ | 35 (12-158) | 105 (24-380) | 0.003 |
| CSF Proteins, mg/dL | 76 (46-153) | 85 (57-167) | 0.26 |
| CSF Glucose, mg/dL | 81 (69-92) | 80 (68-90) | 0.72 |
| Blood glucose, mg/dL | 138 (118-152) | 139 (126-164) | 0.09 |
| Glucose CSF/blood | 0.61 (0.48-0.65) | 0.57 (0.49-0.66) | 0.68 |
| CSF lactate, mEq/L | 3.0 (2.4-3.9) | 3.8 (2.8-5.0) | 0.001 |
| Blood lactate, mEq/L | 1.0 (0.8-1.2) | 1.0 (0.8-1.3) | 0.25 |
| Lactate CSF/blood | 3.0 (2.1-4.1) | 3.6 (2.6-5.0) | 0.005 |
| CGLR | 1.62 (1.15-1.98) | 1.34 (0.80-1.83) | 0.005 |
| Insulin, n (%) | 35 (34) | 31 (27) | 0.38 |
| Noradrenaline, n (%) | 40 (39) | 70 (67) | 0.001 |

CGLR: cerebral spinal fluid glucose to lactate ratio; CSF: cerebral spinal fluid; RBC: red blood cells; WBC: white blood cells; MAP: mean arterial pressure; ICP: intracranial pressure; CPP: cerebral perfusion pressure; PaCO_2_: arterial partial pressure of carbon dioxide; Hb: hemoglobin.

**Supplemental Table S4:** Linear regression model of factors associated with the percentage of change in the cerebral spinal fluid glucose to lactate ratio (ΔCGLR).

|  | **Univariate β-coefficient (95% CI)**  **P value** | **Multivariable β-coefficient (95% CI)**  **P value** |
| --- | --- | --- |
| **Non traumatic vs. traumatic etiology** | 12.98 (-5.83 to 31.80)  p=0.18 | 14.35 (-4.49 to 33.19)  p=0.14 |
| **Interventional group vs. controls** | 21.66 (9.98 to 33.34)  p=0.001 | 27.47 (11.71 to 43.23)  p=0.001 |
| **Glasgow coma scale on admission** | -0.16 (-1.55 to 1.23)  p=0.83 | 0.26 (-1.35 to 1.40)  p=0.97 |
| **CGLR value on baseline** | -3.77 (-12.29 to 4.76)  p=0.39 | 1.03 (-7.69 to 9.75)  p=0.82 |
| **ICP value on baseline** | 0.41 (-0.24 to 1.06)  p=0.21 | -0.40 (-1.28 to 0.47)  p=0.36 |

ICP: intracranial pressure. CGLR: cerebral spinal fluid glucose lactate ratio.

**Supplemental Table S5:** Characteristics of the population according to ICU survival. Data are presented as mean (±SD), median (IQRs) and count (%), as appropriate.

|  | **Survivors**  **(N=164)** | **Non-survivors**  **(N=55)** | **P value** |
| --- | --- | --- | --- |
| Age, years | 56 (±15) | 60 (±11) | 0.03 |
| Male gender, n (%) | 77 (47) | 35 (64) | 0.04 |
| GCS on admission, median (IQR) | 8 (5-13) | 5 (3-10) | 0.001 |
| ***Etiology, n (%)*** | | | 0.36 |
| Subarachnoid Hemorrhage | 89 (54) | 30 (55) |  |
| Intracerebral Hemorrhage | 57 (35) | 16 (29) |  |
| Traumatic Brain Injury | 17 (10) | 8 (15) |  |
| Others | 1 (1) | 1 (2) |  |
| ***Comorbidities, n (%)*** | | | |
| Arterial Hypertension | 86 (55) | 30 (57) | 0.87 |
| Diabetes mellitus | 26 (17) | 7 (13) | 0.67 |
| Heart disease | 21 (14) | 12 (23) | 0.13 |
| COPD | 11 (7) | 6 (11) | 0.38 |
| Liver Cirrhosis | 4 (3) | 4 (8) | 0.12 |
| Chronic Kidney Disease | 6 (4) | 4 (8) | 0.28 |
| Previous neurological disease | 12 (8) | 9 (17) | 0.07 |
| Malignancies | 10 (6) | 6 (11) | 0.24 |
| Immunosuppression | 4 (3) | 2 (4) | 0.99 |
| EVD placement to sample collection, days | 2 (1-3) | 1 (1-3) | 0.79 |
| ***Baseline variables*** | | | |
| ICP, mmHg | 12 (8-18) | 17 (9-24) | 0.007 |
| CPP, mmHg | 84 (74-96) | 82 (72-88) | 0.19 |
| Glucose, mg/dL | 79 (67-90) | 78 (66-89) | 0.56 |
| Lactate, mmol/L | 3.1 (2.6-4.4) | 4.5 (3.5-6.3) | 0.001 |
| CGLR | 1.39 (0.93-1.78) | 0.90 (0.65-1.28) | 0.001 |
| ***After 2 hours*** | | | |
| ICP, mmHg | 10 (7-13) | 12 (7-16) | 0.001 |
| CPP, mmHg | 81 (72-91) | 78 (70-86) | 0.19 |
| Glucose, mg/dL | 80 (69-90) | 81 (69-92) | 0.99 |
| Lactate, mmol/L | 3.0 (2.5-4.4) | 4.0 (3.3-6.0) | 0.001 |
| CGLR | 1.55 (1.03-1.96) | 1.23 (0.69-1.54) | 0.001 |
| Increase in CGLR, n (%) | 101 (62) | 38 (69) | 0.34 |
| Percentage of change in CGLR, median (IQR) | 6.46 (-5.55 to 32.43) | 9.96 (-5.19 to 43.68) | 0.34 |
| ICU length of stay, days | 19 (13-30) | 14 (10-19) | 0.001 |

GCS: Glasgow coma scale; GOS: Glasgow outcome scale; CGLR: cerebral spinal fluid glucose to lactate ratio; CSF: cerebral spinal fluid; RBC: red blood cells; WBC: white blood cells; MAP: mean arterial pressure; ICP: intracranial pressure; CPP: cerebral perfusion pressure; PaCO_2_: arterial partial pressure of carbon dioxide; Hb: hemoglobin; ICU: intensive care unit; EVD: external ventricular drain; COPD: chronic obstructive pulmonary disease.

**Supplemental Table S6:** Characteristics of the population according to the neurological outcome at 3 months. Data are presented as mean (±SD), median (IQRs) and count (%), as appropriate.

|  | **Favorable outcome**  **(N=98)** | **Unfavorable outcome**  **(N=111)** | **P value** |
| --- | --- | --- | --- |
| Age, years | 55 (±15) | 59 (±13) | 0.07 |
| Male gender, n (%) | 50 (47) | 62 (55) | 0.28 |
| GCS on admission | 9 (7-14) | 6 (3-11) | 0.001 |
| **Etiology, n (%)** | | | 0.45 |
| Subarachnoid Hemorrhage | 60 (57) | 59 (52) |  |
| Intracerebral Hemorrhage | 32 (30) | 41 (36) |  |
| Traumatic Brain Injury | 13 (12) | 12 (11) |  |
| Others | 1 (1) | 1 (1) |  |
| ***Comorbidities, n (%)*** | | | |
| Arterial Hypertension | 58 (59) | 58 (52) | 0.33 |
| Diabetes mellitus | 17 (17) | 716(14) | 0.58 |
| Heart disease | 11 (11) | 22 (20) | 0.13 |
| COPD | 5 (5) | 12 (11) | 0.20 |
| Liver Cirrhosis | 0 | 8 (7) | 0.008 |
| Chronic Kidney Disease | 1 (1) | 9 (8) | 0.02 |
| Previous neurological disease | 9 (9) | 12 (11) | 0.82 |
| Malignancies | 10 (6) | 6 (11) | 0.24 |
| Immunosuppression | 3 (3) | 4 (4) | 0.99 |
| EVD placement to sample collection, days | 2 (1-2) | 1 (1-3) | 0.99 |
| ***Baseline variables*** | | | |
| ICP, mmHg | 13 (8-20) | 17 (12-24) | 0.02 |
| CPP, mmHg | 80 (70-90) | 77 (72-86) | 0.11 |
| Glucose, mg/dL | 79 (67-89) | 80 (66-91) | 0.58 |
| Lactate, mmol/L | 3.0 (2.5-4.2) | 4.1 (3.1-5.5) | 0.001 |
| CGLR | 1.41 (0.99-1.92) | 1.05 (0.77-1.51) | 0.001 |
| ***After 2 hours*** | | | |
| ICP, mmHg | 10 (7-14) | 14 (9-18) | 0.03 |
| CPP, mmHg | 83 (74-94) | 80 (72-89) | 0.05 |
| Glucose, mg/dL | 81 (69-89) | 80 (68-92) | 0.88 |
| Lactate, mmol/L | 2.9 (2.2-3.9) | 3.8 (2.9-5.0) | 0.001 |
| CGLR | 1.6 (1.01-1.99) | 1.36 (0.82-1.86) | 0.04 |
| Increase in CGLR, n (%) | 62 (59) | 77 (68) | 0.16 |
| Percentage of change in CGLR, median (IQR) | 7.26 (-5.25 to 34.84) | 9.80 (-4.72 to 44.0) | 0.33 |
| ICU length of stay, days | 18 (12-25) | 18 (12-26) | 0.94 |

GCS: Glasgow coma scale; GOS: Glasgow outcome scale; CGLR: cerebral spinal fluid glucose to lactate ratio; CSF: cerebral spinal fluid; RBC: red blood cells; WBC: white blood cells; MAP: mean arterial pressure; ICP: intracranial pressure; CPP: cerebral perfusion pressure; PaCO_2_: arterial partial pressure of carbon dioxide; Hb: hemoglobin; ICU: intensive care unit; EVD: external ventricular drain; COPD: chronic obstructive pulmonary disease.

**Supplemental Table S7**: Logistic regression of baseline factors associated with ICU mortality. Data are presented as odds ratio (OR) and 95% confidence interval (CI).

|  | **Univariable OR (95% CI)** | **Multivariable OR (95% CI)** |
| --- | --- | --- |
| **Age** | 1.02 (1.0-1.04) | 1.03 (1.01- 1.06) |
| **Glasgow coma scale on admission** | 0.86 (0.80-0.93) | 0.86 (0.78-0.94) |
| **Previous neurological disease** | 2.46 (0.97- 6.21) | 1.55 (0.56-2.31) |
| **Traumatic Brain Injury** | 1.47 (0.60 - 3.63) | 2.28 (0.78-6.68) |
| **Baseline ICP** | 1.05 (1.01-1.08) | 1.03 (0.99-1.07) |
| **Baseline CGLR** | 0.28 (0.15-0.52) | 0.34 (0.18 -0.65) |

ICP: intracranial pressure; CGLR: cerebral spinal fluid glucose to lactate ratio.

**Supplemental Table S8**: Logistic regression of baseline factors associated with unfavorable neurological outcome at 3 months. Data are presented as odds ratio (OR) and 95% confidence interval (CI).

|  | **Univariable OR (95% CI)** | **Multivariable OR (95% CI)** |
| --- | --- | --- |
| **Age** | 1.02 (1.0-1.04) | 1.03 (1.01- 1.05) |
| **Glasgow coma scale on admission** | 0.86 (0.80-0.93) | 0.85 (0.79-0.91) |
| **Traumatic Brain Injury** | 0.85 (0.37-1.96) | 1.04 (0.39 – 2.75) |
| **Chronic kidney failure** | 8.56 (1.06-68.83) | 9.38 (1.05-84.08) |
| **ICP** | 1.04 (1.01-1.07) | 1.03 (0.99-1.06) |
| **CGLR** | 0.28 (0.15-0.52) | 0.47 (0.29 -0.75) |

ICP: intracranial pressure; CGLR: cerebral spinal fluid glucose to lactate ratio.

**Supplemental Figure S1**: Receiver operator curve (ROC) of baseline cerebral spinal fluid glucose to lactate ratio (CGLR) to predict hospital mortality. Area under the ROC: 0.73 (95% CI 0.623-0.782).


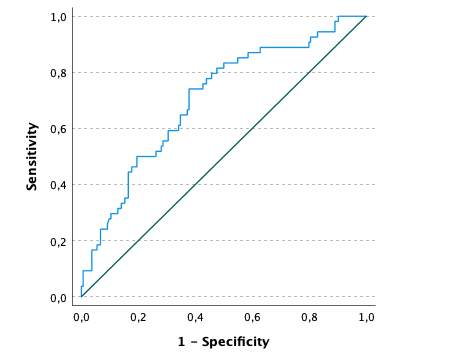


**Supplemental Figure S2**: Receiver operator curve (ROC) of baseline cerebral spinal fluid glucose to lactate ratio (CGLR) ability to predict poor outcome at 3 months. Area under the ROC: 0.66 (0.59-0.74).


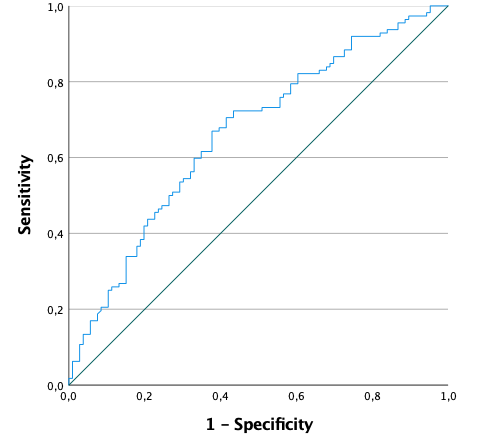


**Supplemental Figure S3:** Changes over time of CGLR between study groups in traumatic brain injury patients (n=25)

P values represent the comparison of trend over time in the two groups (time-group interaction) and were calculated using a mixed model.


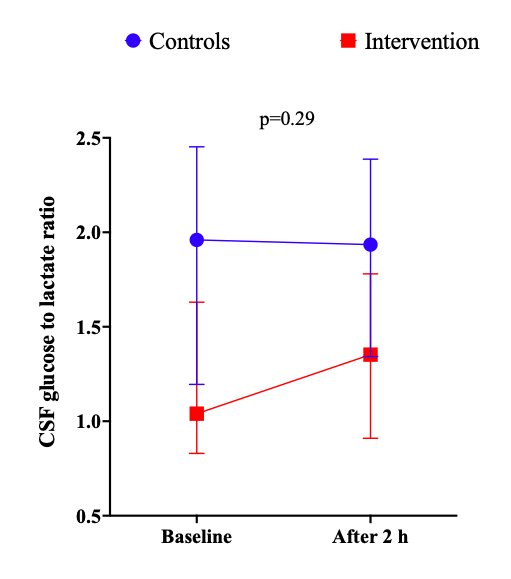


**Supplemental Figure S4:** Changes over time of CGLR between study groups in intracerebral hemorrhage patients (n=73)

P values represent the comparison of trend over time in the two groups (time-group interaction) and were calculated using a mixed model.


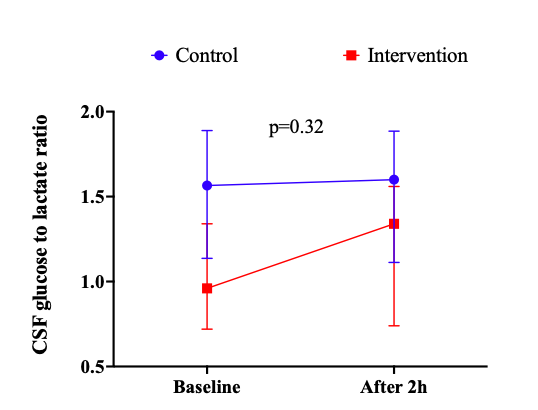


**Supplemental Figure S5:** Changes over time of CGLR between study groups in subarachnoid hemorrhage patients (n=119).

P values represent the comparison of trend over time in the two groups (time-group interaction) and were calculated using a mixed model.


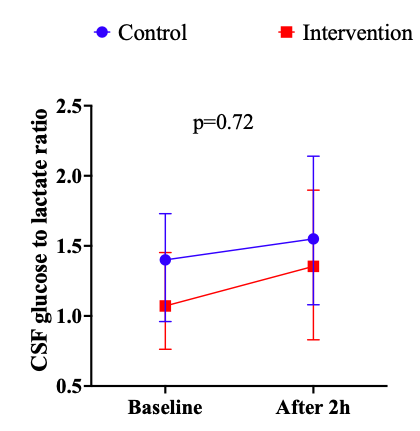

Supplement: Supplementary file 1 — Additional file 1. Supplemental electronic material of STAR-TRIP study. [file 13054_2023_4409_MOESM1_ESM.docx]
